# Effect of carotenoids dietary supplementation on macular function in diabetic patients

**DOI:** 10.1186/s40662-017-0088-4

**Published:** 2017-10-15

**Authors:** Marilita M. Moschos, Maria Dettoraki, Michael Tsatsos, George Kitsos, Christos Kalogeropoulos

**Affiliations:** 1 First Department of Ophthalmology, Medical School, National and Kapodistrian University of Athens, 6 Ikarias street, Ekali, 14578 Athens, Greece; 2 Department of Ophthalmology, Medical School, Aristotle University of Thessaloniki, Thessaloniki, Greece; 3 Department of Ophthalmology, Medical School, University of Ioannina, Ioannina, Greece

**Keywords:** Carotenoids, Diabetes mellitus, Visual function, Multifocal electroretinography, Optical coherence tomography

## Abstract

**Background:**

Diabetic retinopathy is a major cause of visual impairment and blindness among working-age people worldwide. The aim of our study was to investigate the effects of a carotenoid supplementation on retinal thickness and macular function of patients with diabetes using optical coherence tomography (OCT) and multifocal electroretinography (mfERG).

**Methods:**

A retrospective study of one hundred and twenty eyes of sixty patients age between 40 and 60 years with non-insulin dependent type 2 diabetes mellitus without diabetic retinopathy who underwent OCT and mfERG and took vitamin supplements for a period of two years. Patients received a carotenoid supplement containing lutein (10 mg), zeaxanthin (2 mg) and meso-zeaxanthin (10 mg) once a day for two years. The thickness of the fovea was evaluated using OCT and the macular function was tested by mfERG.

**Results:**

OCT showed an increase in the central foveal thickness and mfERG revealed increased retinal response density within the central 13° surrounding the fovea (rings 1 to 3) at two years after the onset of carotenoids supplement intake.

**Conclusion:**

The use of carotenoid supplements may be of benefit for improving visual function of type 2 diabetes patients. However, further study is needed to assess the treatment’s long-term efficacy.

## Background

Diabetic retinopathy (DR) is a major cause of visual impairment and blindness among working-age people worldwide. Despite studies showing that timely treatment of DR can significantly reduce the risk of visual complications and the advances in clinical management of the disease, DR visual impairment increased by an alarming 64% over the last two decades globally [1]. Diabetic macular oedema (DME) is one of the major complications of DR and the most common form of sight-threatening retinopathy in diabetes affecting more than 20 million people worldwide [2, 3]. Poor glycaemic and blood pressure control are associated with the presence and development of the disease. The current treatment options of DME include laser photocoagulation, intravitreal injections of anti-vascular endothelial growth factor (anti-VEGF) agents and corticosteroids.

There have been few human studies evaluating the effects of dietary supplements on the occurrence and progression of DR. Although the positive influence of a nutritional supplement on the progression of a vision-threatening eye disease, age-related macular degeneration (AMD), has been demonstrated by the Age-Related Eye Disease Study (AREDS), the available evidence in support of the use of carotenoids for retinal health in DR is scant [4]. Daily consumption of a multi-component formula containing xanthophyll pigments, antioxidants, and selected botanical extracts had been shown to improve contrast sensitivity, macular pigment optical density, colour discrimination and perimetry in patients with diabetes, both with and without retinopathy [5]. Furthermore, higher concentrations of the plasma carotenoids lutein, zeaxanthin and lycopene, which are largely dependent on dietary intake, are considered to have a protective role against DR [6]. Lutein and zeaxanthin intake has been reported to improve visual acuity, contrast sensitivity and macular oedema in patients with non-proliferative DR [7].

The carotenoids lutein, zeaxanthin and meso-zeaxanthin accumulate in the central retina, where they are collectively known as macular pigment. Lutein is the dominant carotenoid in the peripheral macula, zeaxanthin in the mid-peripheral macula, and meso-zeaxanthin at the epicentre of the macula [8, 9]. In the last decade, the macular pigment has generated increased interest due to its possible protective role against AMD, which may be attributed to its antioxidant effects and protection of the retina against the phototoxic activity of blue light [10–13]. Humans cannot synthesize macular pigment but absorb lutein and zeaxanthin from the diet, mainly the fruits, vegetables and egg yolks.

Multifocal electroretinography (mfERG) has been evaluated as an objective, non-invasive method to detect subclinical DR and assess changes in the retinal function of diabetic patients [14–17] Moreover, mfERG allows a topographic mapping of retinal dysfunction in DR [18]. MfERG reflects not only the electrophysiological responses of the photoreceptors but also those of the inner retinal layers, including bipolar cells and Muller cells, which are mainly affected by DR [19].

The aim of our study was to investigate the effects of a carotenoid supplement (Macushield), containing lutein, zeaxanthin, and meso-zeaxanthin, on retinal thickness and macular function of patients with type 2 diabetes, using OCT and mfERG. To the best of our knowledge, the use of mfERG to evaluate the effects of food supplements on retinal function of diabetic patients has not been reported.

## Methods

### Subjects

Sixty patients were included in this retrospective study who received a soft gel capsule containing lutein (10 mg), zeaxanthin (2 mg) and meso-zeaxanthin (10 mg) in a sunflower oil suspension (commercially available as Macushield). Patients were instructed to take one capsule daily with a meal. The data collected include best corrected visual acuity (BCVA), central foveal thickness (CFT) and mfERG responses measured at baseline (pre-medication) and after two years of carotenoid supplementation. BCVA was measured with the use of a Snellen chart.

Inclusion criteria included BCVA ≥8/10 in each eye, normal colour vision test and no signs of DR. All patients were treated with oral antidiabetic therapy. No previous lutein and/or other anti-oxidants supplementation were taken. Patients with diabetes were excluded if they exhibited any ocular disease such as cataract, glaucoma, age-related macular degeneration, myopia of more than 6 D or had previously undergone cataract extraction or any treatment for DR or diabetic maculopathy.

The study was conducted at the 1st Department of Ophthalmology, “G. Gennimatas” General Hospital, University of Athens, Athens, Greece and adhered to the principles laid out in the Declaration of Helsinki. The study was approved by the institutional review board of “G. Gennimatas” General Hospital, Athens, Greece.

### SD-OCT scan acquisition and analysis

Sectional images of the macula of each patient were scanned using SD-OCT (Spectralis OCT, Heidelberg Engineering, Heidelberg, Germany) at baseline and 2 years after carotenoid supplement intake. The patients were asked to gaze at the fixation light within the instrument and the foveolar fixation was confirmed by observing the retinal image through the infrared monitoring camera. A 9-mm line scan along the horizontal meridian centered at the fovea was obtained using high-resolution settings. The line scan was obtained as an average of 100 scans to give the highest signal-to-noise ratio (>25 dB).

The foveal line scans were examined for local abnormalities such as macular oedema. The scans were then analysed using a computer-aided, manual technique for the measurement of central foveal thickness (CFT, the distance between the innermost border of the retina towards the vitreous to the ellipsoid zone) in all patients.

### mfERG recording

MfERG was performed according to the guidelines by the International Society for Clinical Electrophysiology of Vision using the VERIS III (Visual Evoked Response Imaging System; Tomey, Nagoya, Japan) [20]. MfERG was recorded with an active fibre electrode positioned on the bulbar conjunctiva directly beneath the cornea and with a reference inactive electrode attached to the skin, near to the orbital rim and lateral to the corresponding eye. The ground electrode was attached to the earlobe. The active, inactive and ground electrodes were connected to a junctional box, from which the signals were delivered to additional recording components for amplification and display. The recording was performed with eyes corrected for near vision. Pupils were fully dilated with topical 0.5% tropicamide and 5% phenylephrine eye drops. The fellow eye was closed and the duration of the data acquisition was four minutes divided into eight sessions of 30 s. Multiple retinal areas were stimulated simultaneously using a stimulus array of 61 hexagons displayed on a cathode ray tube (CRT) monitor (Sony, Tokyo). Each hexagon was independently alternated between black and white at a rate of 75 Hz and the stimulation technique allowed a retinal response from each stimulus. A red fixation point of 2 mm diameter was used. The stimulus luminance was 200 cd/m^2^ for the bright flashes and 1 cd/m^2^ for the dark flashes. The radius of the stimulus array subtended approximately 20° high and 25° wide. The bandwidth of the amplifier was 10–300 Hz, and the amplification was ×10000. Topical anaesthesia with 0.5% proparacaine hydrochloride eye drops was installed before the recording. The recording procedure was repeated when spurious potentials from eye blinks or ocular movements were recorded.

The mfERG stimuli location and anatomical areas corresponded roughly to five concentric rings as follows: ring 1 to the fovea (0°-2°), ring 2 to the parafovea (2°-7°), ring 3 to the perifovea (7°-13°), ring 4 to the near periphery (13°-22°) and ring 5 to the central part of the middle periphery (22°-30.5°). The retinal response density (RRD, amplitude per unit retinal area, nV/deg^2^) and the implicit time (P_1_ latency, ms) of the first positive peak of each individual ring were measured in each patient at baseline and 2 years after the carotenoid supplement intake.

#### Statistical analysis

The Gaussian distribution assumption was tested using the method of Kolmogorov and Smirnov. All variables succeeded in passing the normality test. For statistical analysis, the paired comparison t-test was used to test the significance of the mean values before and after the supplement intake in diabetic patients. The data were expressed as the mean ± standard deviation (SD). A *p* value of less than 0.05 was considered to indicate significance.

## Results

The study included 120 eyes of 60 patients with type 2 diabetes. The mean age of patients at baseline was 50 years with a range from 40 to 60 years. Of the 60 patients, 31 (52%) were males and 29 (48%) were females.

Two years after the carotenoid supplementation, BCVA remained normal (≥ 9/10) in each eye. The mean CFT in the right eye increased from 157.4 ± 13.7 μm at baseline to 162.8 ± 13.1 μm following carotenoid intake (*p* < 0.001). Similarly, in the left eye, the mean CFT increased from 157.1 ± 14 μm to163.4 ± 13.2 μm after carotenoid intake (p < 0.001) (Table 1). No intraretinal fluid or cystic changes in the retina were detected on morphological analysis of the OCT cross-sectional scans in any of the eyes examined.

**Table 1:** Optical coherence tomography (OCT) and multifocal electroretinography (mfERG) findings before and two years after the carotenoids supplementation in diabetic patients

Parameter	Before	After	*p* value
CFT (μm)			
OD	157.4 ± 13.7	162.8 ± 13.1	<0.001
OS	157.1 ± 14.0	163.4 ± 13.2	<0.001
Retinal Response Density (nV/deg^2^)			
Ring 1 OD	172.1 ± 18.4	188.9 ± 17.2	<0.001
Ring 1 OS	171.4 ± 18.7	187.9 ± 16.6	<0.001
Ring 2 OD	65.2 ± 9.0	75.7 ± 9.7	<0.001
Ring 2 OS	65.8 ± 9.2	76.3 ± 9.1	<0.001
Ring 3 OD	42.3 ± 5.0	45.4 ± 4.5	<0.001
Ring 3 OS	42.4 ± 4.0	46 ± 4.1	<0.001
Latency (ms)			
Ring 1 OD	42.9 ± 1.7	42.7 ± 1.5	0.11
Ring 1 OS	42.8 ± 1.5	42.8 ± 1.5	0.62
Ring 2 OD	39.9 ± 1.7	39.9 ± 1.8	0.53
Ring 2 OS	39.4 ± 1.9	39.5 ± 1.9	0.42
Ring 3 OD	34.4 ± 0.8	34.3 ± 1.0	0.69
Ring 3 OS	34.3 ± 0.8	34.2 ± 0.9	0.42

*CFT*=central foveal thickness; *OD*=oculus dexter; *OS*=oculus sinister

Data are expressed as mean ± SD

**Fig. 1:**
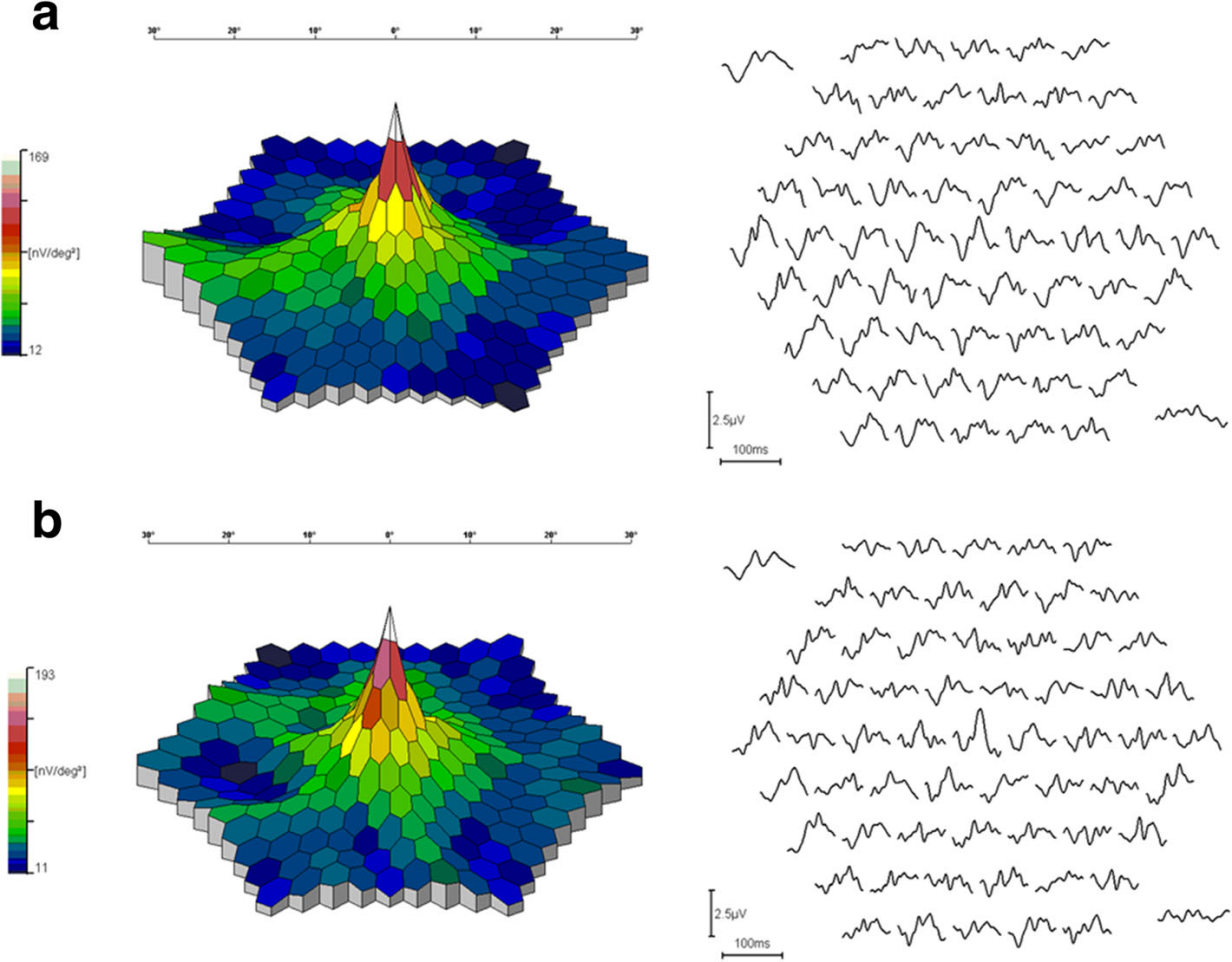
The three-dimensional topography of mfERG and the ERG traces in a case with type 2 diabetes without DR (**a**) before and (**b**) two years after the carotenoid supplementation. An improvement in the three-dimensional appearance of the mfERG and ERG traces at the central area was found at two years after the carotenoid supplementation

The RRD of mfERG significantly increased in all central 3 rings in both eyes of the patients two years after supplement intake compared to baseline, as shown in Table 1. No differences in P1 latency were observed in any of the 3 central rings in both eyes after supplement intake compared to baseline (Fig. 1).

No adverse reactions due to the supplement intake were recorded during the entire period of treatment.

## Discussion

This study demonstrated that carotenoid supplements increased the CFT on OCT and significantly improved the RRD on mfERG at the central rings in patients with type 2 diabetes without DR, suggesting that carotenoids may have a beneficial effect on the macular function of diabetic patients.

Several studies have shown that serum lutein and zeaxanthin concentrations are significantly lower in patients with non-proliferative DR than those in normal subjects [6, 7]. Possible reasons include an unhealthy diet poor in fruits and vegetables since the diabetic patients’ body mass index is generally higher than the normal standard, decreased absorption of lutein and zeaxanthin because of hyperglycaemia and limited accumulation in the retina due to destructing local microcirculation characteristic in DR [7]. Following dietary supplementation of lutein and zeaxanthin in diabetic patients, their serum concentrations are higher than those in normal subjects [7, 21]. Specifically, serum lutein can reach maximum concentration 16 h after 10 mg of lutein intake [22]. Furthermore, several studies have demonstrated that following serum lutein and zeaxanthin elevation, the lutein, and zeaxanthin density in the retina also increases [6, 21, 23, 24]. Therefore, a positive relationship exists between the higher consumption of these carotenoids, the higher serum carotenoid levels, and the higher macular pigment density.

Brazionis et al. assessed the relationship between the major carotenoids, lutein, zeaxanthin and lycopene, and DR in a cross-sectional study of 111 individuals with type 2 diabetes [6]. The authors reported that a higher combined lutein, zeaxanthin and lycopene concentration in plasma was associated with significantly lower odds of DR, after adjusting for potential confounding retinopathy risk factors. Hu et al. reported a decrease in the average foveal thickness of 30 diabetic patients with non-proliferative DR and macular oedema three months after lutein and zeaxanthin supplementation compared to pre-medication status [7]. The authors proposed that the carotenoids may play a role in diabetic macular oedema since lutein and zeaxanthin can reduce vascular permeability, inhibit vascular leakage and protect the integrity of blood vessels.

Loss of retinal capillaries leading to progressive retinal hypoxia, increased retinal vascular permeability, and new retinal vessel growth are characteristic of DR [25]. Moreover, DR is considered a multifactorial disease with various abnormalities contributing to its development. Oxidative stress is increased in the retina of diabetic patients and is implicated in the development of DR. DR is also known to have an inflammatory component in that leukostasis occurs and increased levels of the adhesion molecule ICAM-1 have been found in the retina of diabetics [26]. VEGF, an angiogenic factor that induces vascular endothelial cell proliferation, migration, and vasopermeability in many tissues, is also elevated in the retina and vitreous of diabetic patients and is implicated in the pathogenesis of DR [25]. Studies have shown that lutein can attenuate oxidative stress in experimental models of early DR and zeaxanthin significantly inhibits diabetes-induced retinal oxidative damage and elevations in VEGF and adhesion molecule ICAM-1 in diabetic rats [27, 28]. Hence, animal studies have demonstrated that carotenoids have a protective role against the abnormalities associated with the pathogenesis of DR.

Studies in patients with AMD and healthy subjects have evaluated the impact of the same carotenoid formulation as in our study on macular function. Macular pigment optical density and contrast sensitivity have been found to increase after the carotenoid supplementation, whereas no significant changes in BCVA or progression to advanced AMD were observed in healthy subjects and patients with early AMD [12, 13, 29]. A recent report demonstrated that the daily oral supplementation with Macushield for six months in patients with retinal pathology (including those with DR) increased the mean macular pigment optical density and significantly increased the contrast sensitivity at low and medium spatial frequencies [30]. Furthermore, a significant improvement in the vision-related quality of life was reported. Recently, the Central Retinal Enrichment Supplementation Trial (CREST) study reported a significant increase in contrast sensitivity of subjects free of retinal disease after the daily consumption of the same formulation used in the previous studies compared to placebo [29]. In our study, two years after the daily supplementation with Macushield in patients with type 2 diabetes, mfERG macular responses significantly improved and foveal thickness increased compared to baseline findings, indicating an improvement of visual function. Visual acuity did not change over the study period, which is consistent with the CREST study as well as with the studies concerning patients with early AMD [13, 29].

Changes in retinal function can be depicted in detail by means of mfERG recording, which is essential mainly for the investigation of macular lesions. It has been shown that in all types of maculopathy there is a decrease or loss of central electrical response, which is related to the degree and extent of the lesion. MfERG is a valuable method particularly in cases where the patient is visually asymptomatic, visual acuity is normal and macular lesions are not clearly visible ophthalmoscopically [31]. MfERG reflects the electrophysiological responses mainly from the inner retinal layers. DR is characterized by the pathology of the microvasculature in the inner nuclear layer, where the primary generators of mfERG, the bodies of bipolar cells, are located. Moreover, changes in the retinal vasculature of diabetic subjects have been reported to take place before retinopathy becomes clinically apparent [32]. Thus, mfERG is a suitable method for the study of DR. Several investigators have used mfERG recordings to assess retinal dysfunction both in patients with DR [14, 15, 17, 18] and in diabetic subjects without signs of retinopathy [15, 16, 18, 33]. A delay in the implicit time of the first order component of mfERG has been demonstrated in diabetics without retinopathy compared to normal subjects, whereas in patients with DR both a decrease in amplitude and a delay in peak implicit time of mfERG has been reported. Harrison et al. have shown that the implicit time measure of mfERG is a good predictor for the development of retinopathy in adult patients with diabetes in a 1-year follow-up period [33]. Patients with type 1 diabetes display a greater risk for the onset of retinopathy with smaller comparative delays in implicit time than the type 2 group.

In our study, the central foveal thickness of patients with diabetes and no retinopathy measured with the use of OCT increased at two years after the carotenoid supplementation compared with the baseline values. It should be mentioned, however, that several studies have suggested that patients with diabetes and no retinopathy have retinal thickness values that are similar to values from populations without diabetes and normal retinas [34, 35]. Further studies are needed to assess the significance of the anatomical change demonstrated by OCT in our study. Nevertheless, until now, no previous study evaluating the effects of nutritional supplements on diabetes has utilized mfERG as a visual function evaluation tool. Limitations of our study include its retrospective nature, the absence of a placebo group and the non-assessment of serum carotenoids and macular pigment optical density in diabetic patients.

## Conclusions

Our study confirms the effectiveness of carotenoid intake on visual function of type 2 diabetic patients. Furthermore, the present study gives strength to the recommendation of increasing consumption of lutein and zeaxanthin-rich foods. Further prospective studies are needed to determine whether both over time and in more severe stages of DR a high concentration of carotenoids can delay the onset or reduce the risk of progression in DR.

## Abbreviations

AMD: Age-related macular degeneration
anti-VEGF: Anti-vascular endothelial growth factor
AREDS: Age-Related Eye Disease Study
BCVA: Best corrected visual acuity
CFT: Central foveal thickness,
CREST: Central Retinal Enrichment Supplementation Trial
DME: Diabetic macular oedema
DR: Diabetic retinopathy
mfERG: Multifocal electroretinography
RRD: Retinal response density
SD-OCT: Spectral domain optical coherence tomography

## References

[1] Leasher JL, Bourne RR, Flaxman SR, Jonas JB, Keeffe J, Naidoo K (2016). Global Estimates on the Number of People Blind or Visually Impaired by Diabetic Retinopathy: A Meta-analysis From 1990 to 2010. Diabetes Care.

[2] Gundogan FC, Yolcu U, Akay F, Ilhan A, Ozge G, Uzun S (2016). Diabetic Macular Edema. Pak J Med Sci.

[3] Tan GS, Cheung N, Simó R, Cheung GC, Wong TY (2016). Diabetic macular oedema. Lancet Diabetes Endocrinol..

[4] Age-Related Eye Disease Study Research Group (2001). A randomized, placebo-controlled, clinical trial of high-dose supplementation with vitamins C and E, beta carotene, and zinc for age-related macular degeneration and vision loss: AREDS report no. 8. Arch Ophthalmol.

[5] Chous AP, Richer SP, Gerson JD, Kowluru RA (2016). The Diabetes Visual Function Supplement Study (DiVFuSS). Br J Ophthalmol.

[6] Brazionis L, Rowley K, Itsiopoulos C, O'Dea K (2009). Plasma carotenoids and diabetic retinopathy. Br J Nutr.

[7] Hu BJ, Hu YN, Lin S, Ma WJ, Li XR (2011). Application of Lutein and Zeaxanthin in nonproliferative diabetic retinopathy. Int J Ophthalmol..

[8] Bone RA, Landrum JT, Friedes LM, Gomez CM, Kilburn MD, Menendez E (1997). Distribution of lutein and zeaxanthin stereoisomers in the human retina. Exp Eye Res.

[9] Nolan JM, Meagher K, Kashani S, Beatty S (2013). What is meso-zeaxanthin, and where does it come from?. Eye (Lond).

[10] Bone RA, Landrum JT, Hime GW, Cains A, Zamor J (1993). Stereochemistry of the human macular carotenoids. Invest Ophthalmol Vis Sci.

[11] Landrum JT, Bone RA (2001). Lutein, zeaxanthin, and the macular pigment. Arch Biochem Biophys.

[12] Ma L, Liu R, Du JH, Liu T, Wu SS, Liu XH. Lutein, Zeaxanthin and Meso-zeaxanthin Supplementation Associated with Macular Pigment Optical Density. Nutrients. 2016;8(7). doi: 10.3390/nu8070426. 10.3390/nu8070426 PMC4963902 27420092

[13] Akuffo KO, Nolan JM, Howard AN, Moran R, Stack J, Klein R (2015). Sustained supplementation and monitored response with differing carotenoid formulations in early age-related macular degeneration. Eye (Lond)..

[14] Ng JS, Bearse MA Jr, Schneck ME, Barez S, Adams AJ. Local diabetic retinopathy prediction by multifocal ERG delays over 3 years. Invest Ophthalmol Vis Sci. 2008;49:1622–8. 10.1167/iovs.07-1157 18385083

[15] Palmowski AM, Sutter EE, Bearse MA, Fung W (1997). Mapping of retinal function in diabetic retinopathy using the multifocal electroretinogram. Invest Ophthalmol Vis Sci..

[16] Tyrberg M, Lindblad U, Melander A, Lövestam-Adrian M, Ponjavic V, Andréasson S (2011). Electrophysiological studies in newly onset type 2 diabetes without visible vascular retinopathy. Doc Ophthalmol..

[17] Yu M, Zhang X, Zhong X, Yu Q, Jiang F, Ma J, et al. Multifocal electroretinograms in the early stages of diabetic retinopathy. Chin Med J (Engl). 2002;115:563–6. 12133299

[18] Abdelkader M (2013). Multifocal electroretinogram in diabetic subjects. Saudi J Ophthalmol.

[19] Kondo M, Miyake Y, Horiguchi M, Suzuki S, Tanikawa A (1995). Clinical evaluation of multifocal electroretinogram. Invest Ophthalmol Vis Sci.

[20] Hood DC, Bach M, Brigell M, Keating D, Kondo M, Lyons JS (2012). ISCEV standard for clinical multifocal electroretinography (mfERG) (2011 edition). Doc Ophthalmol.

[21] Curran-Celentano J, Hammond BR Jr, Ciulla TA, Cooper DA, Pratt LM, Danis RB. Relation between dietary intake, serum concentrations, and retinal concentrations of lutein and zeaxanthin in adults in a Midwest population. Am J Clin Nutr. 2001;74:796–802. 10.1093/ajcn/74.6.796 11722962

[22] Kostic D, White WS, Olson JA (1995). Intestinal absorption, serum clearance, and interactions between lutein and beta-carotene when administered to human adults in separate or combined oral doses. Am J Clin Nutr.

[23] Bone RA, Landrum JT, Guerra LH, Ruiz CA (2003). Lutein and zeaxanthin dietary supplements raise macular pigment density and serum concentrations of these carotenoids in humans. J Nutr.

[24] Burke JD, Curran-Celentano J, Wenzel AJ (2005). Diet and serum carotenoid concentrations affect macular pigment optical density in adults 45 years and older. J Nutr.

[25] Aiello LP, Wong JS (2000). Role of vascular endothelial growth factor in diabetic vascular complications. Kidney Int Suppl.

[26] Joussen AM, Poulaki V, Le ML, Koizumi K, Esser C, Janicki H (2004). A central role for inflammation in the pathogenesis of diabetic retinopathy. FASEB J.

[27] Kowluru RA, Menon B, Gierhart DL (2008). Beneficial effect of zeaxanthin on retinal metabolic abnormalities in diabetic rats. Invest Ophthalmol Vis Sci.

[28] Muriach M, Bosch-Morell F, Alexander G, Blomhoff R, Barcia J, Arnal E (2006). Lutein effect on retina and hippocampus of diabetic mice. Free Radic Biol Med.

[29] Nolan JM, Power R, Stringham J, Dennison J, Stack J, Kelly D (2016). Enrichment of Macular Pigment Enhances Contrast Sensitivity in Subjects Free of Retinal Disease: Central Retinal Enrichment Supplementation Trials-Report 1. Invest Ophthalmol Vis Sci.

[30] Crosby-Nwaobi R, Hykin P, Peto T, Sivaprasad S (2016). An exploratory study evaluating the effects of macular carotenoid supplementation in various retinal diseases. Clin Ophthalmol.

[31] Dettoraki M, Moschos MM (2016). The Role of Multifocal Electroretinography in the Assessment of Drug-Induced Retinopathy: A Review of the Literature. Ophthalmic Res.

[32] Cai J, Boulton M (2002). The pathogenesis of diabetic retinopathy: old concepts and new questions. Eye (Lond)..

[33] Harrison WW, Bearse MA, Ng JS, Jewell NP, Barez S, Burger D (2011). Multifocal electroretinograms predict onset of diabetic retinopathy in adult patients with diabetes. Invest Ophthalmol Vis Sci..

[34] Massin P, Erginay A, Haouchine B, Mehidi AB, Paques M, Gaudric A (2002). Retinal thickness in healthy and diabetic subjects measured using optical coherence tomography mapping software. Eur J Ophthalmol.

[35] Bressler NM, Edwards AR, Antoszyk AN, Beck RW, Browning DJ, Ciardella AP (2008). Retinal Thickness on Stratus Optical Coherence Tomography in People with Diabetes and Minimal or No Diabetic Retinopathy. Am J Ophthalmol.

